# Correspondence to *European Heart Journal—Quality of Care and Clinical Outcomes* in response to paper by Thomas, M. *et al.* 2021: Predicting the EQ-5D from the Kansas City Cardiomyopathy Questionnaire (KCCQ) in patients with heart failures

**DOI:** 10.1093/ehjqcco/qcab033

**Published:** 2021-04-29

**Authors:** Hasnain Dalal, Rod S Taylor, John G Cleland

**Affiliations:** 1 Primary Care Research Group, University of Exeter, Exeter, UK; 2 Knowledge Spa, Royal Cornwall Hospitals NHS Trust Truro TR1 3HD, UK; 3 MRC/CSO Social and Public Health Sciences Unit & Robertson Centre for Biostatistics, University of Glasgow, Glasgow, UK

We congratulate Thomas *et al.*^1^ for developing algorithms mapping Kansas CityCardiomyopathy Questionnaire (KCCQ) toEQ-5D health-utility scores for patients with heart failure (HF). EQ-5D is a standard tool for assessing cost-effectiveness (QALYs) across disease areas. However, such generic health-utility measures may fail to capture key health states relevant to heart failure (such as breathlessness and fatigue). There is a need fora disease-specific utility measure for heart failure.^2^

Mapping disease-specific, patient-reported outcomes like KCCQ to EQ-5D has limitations, as the authors acknowledge.^1^ However, the potential insensitivity of EQ-5D to changes in health state should be considered.^2–4^ EQ-5D may be sensitive to the effects of interventions in advanced heart failure (New York Heart Association (NYHA) III–IV), but perhaps less so for milder disease.^4^^,^^5^

Thomas *et al*. used EuroQoL-5 Dimension (EQ-5D) data from the HF-ACTION trial (*n* = 2331 HF patients) but do not mention that no difference was observed at 12 months in either EQ-5D index score or visual analogue scale (VAS) with exercise-based rehabilitation compared with control (VAS: Rehab: 1 ± 17 vs. control: 2 ± 17; *P* = 0.15).^3^ Was the intervention ineffective or was the tool insensitive to change? Mapping KCCQ to a tool that is not sensitive to change could undervalue the effects of the intervention.

**Conflict of interest:** none declared.
